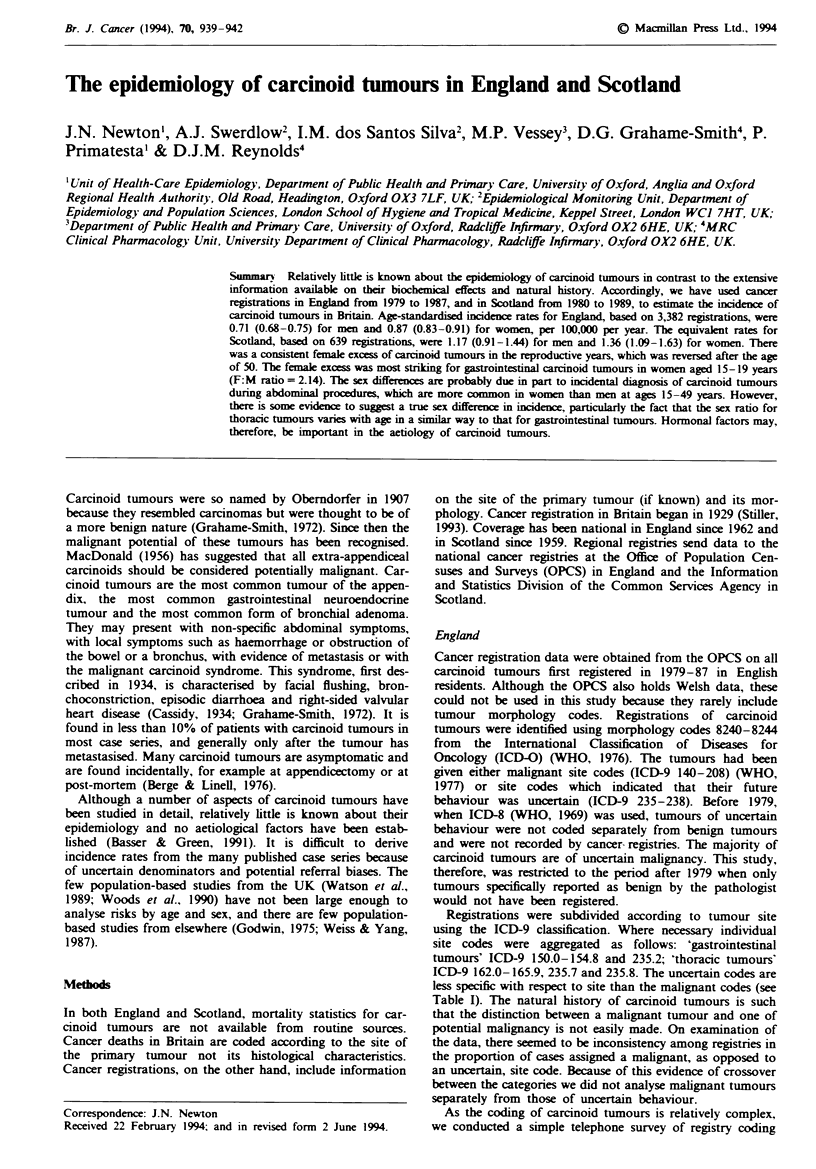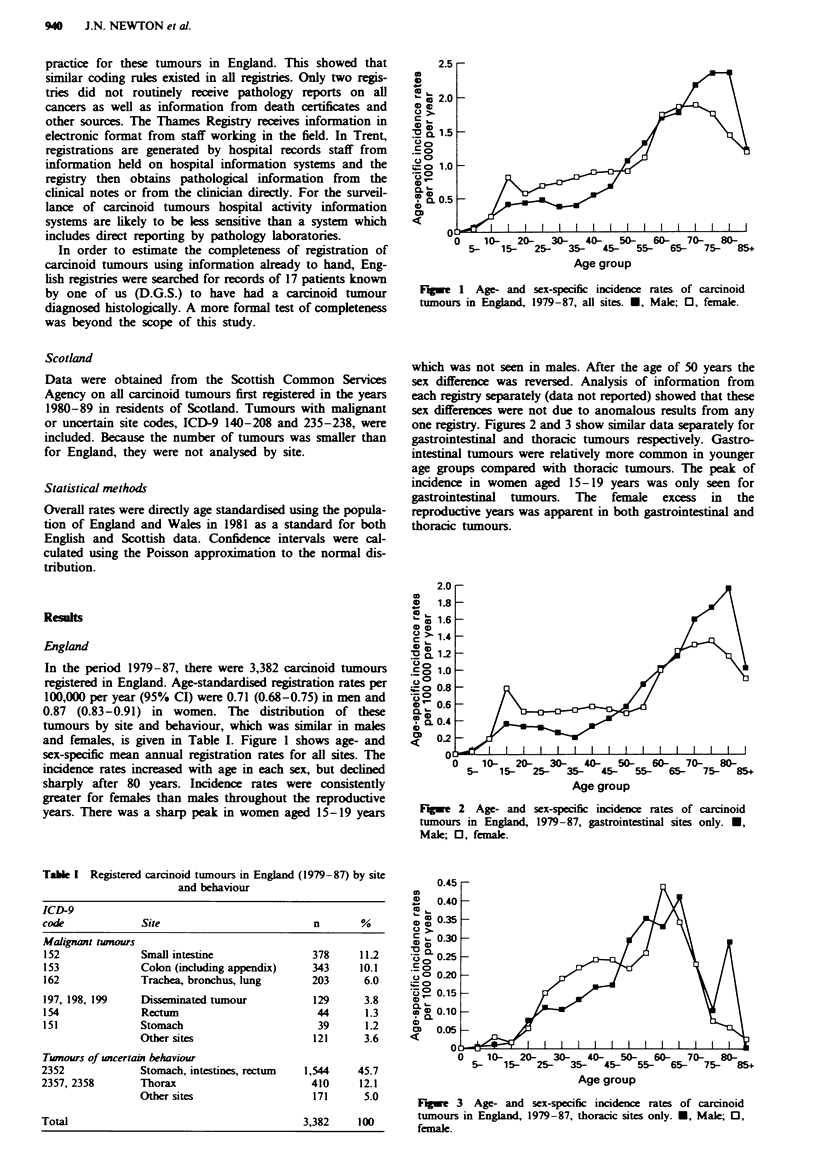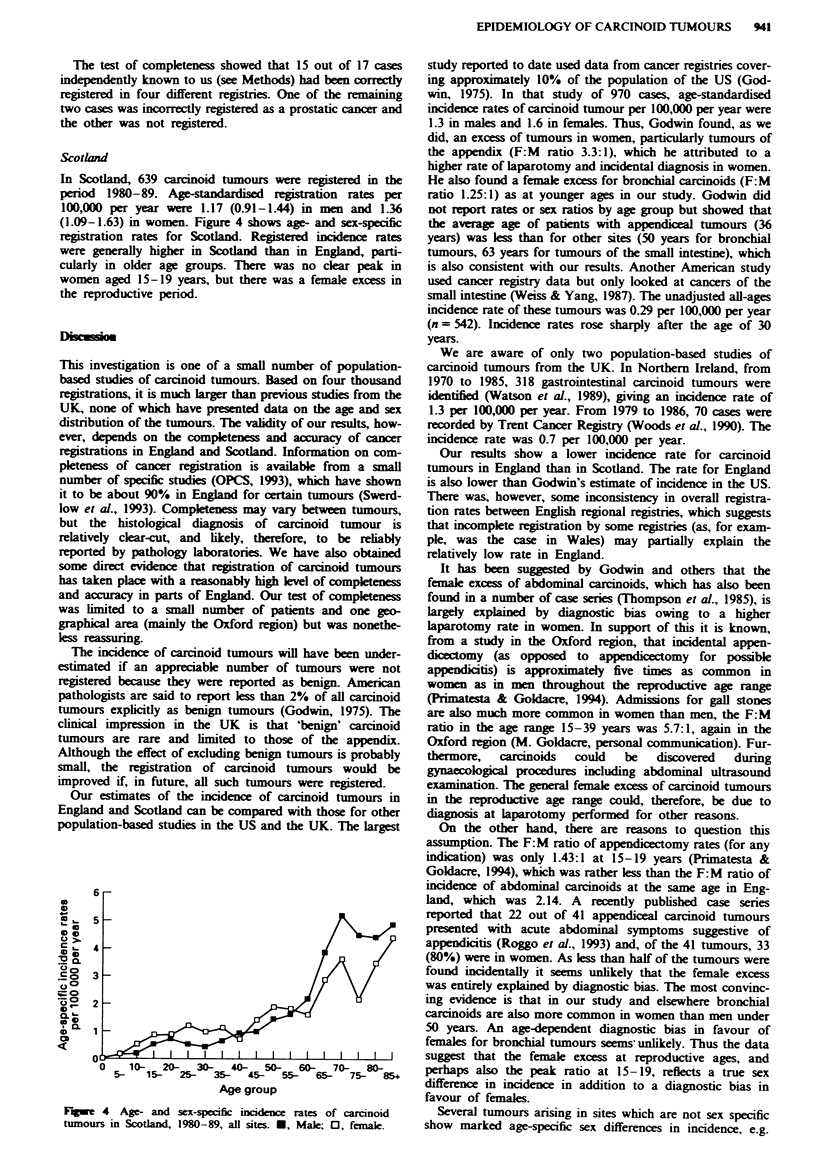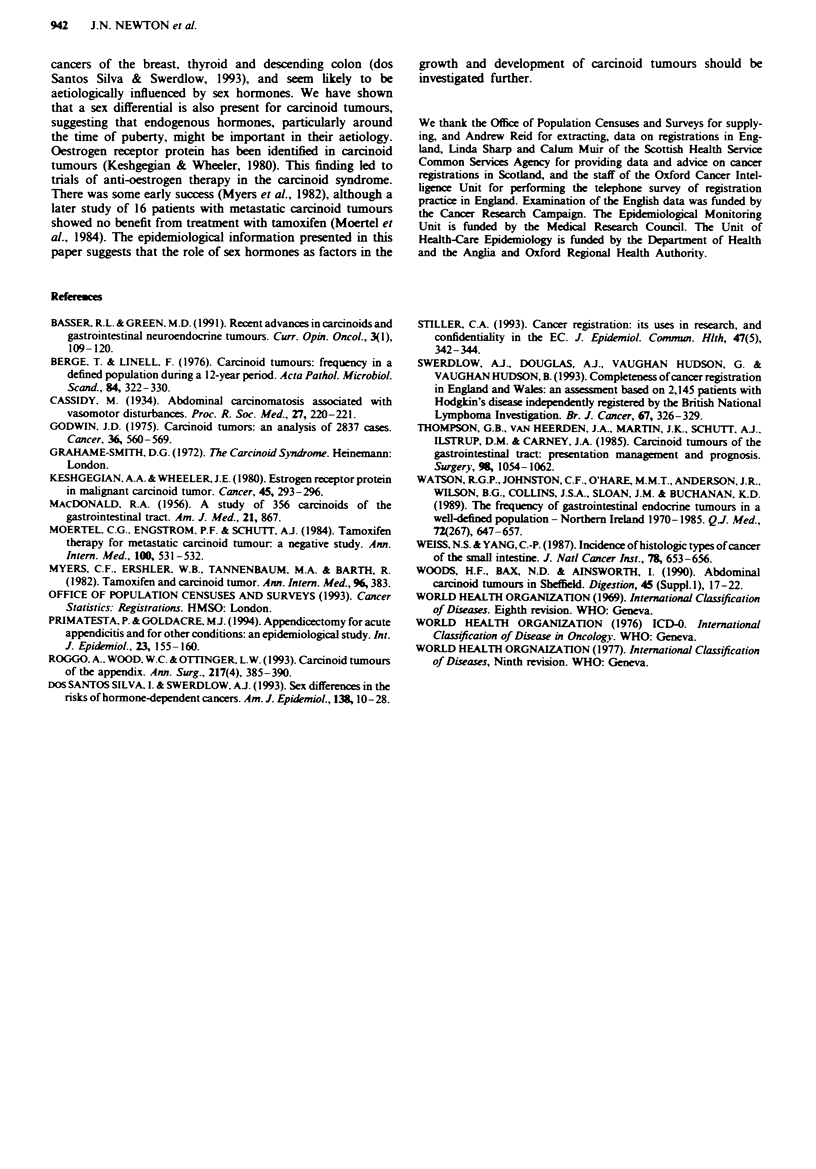# The epidemiology of carcinoid tumours in England and Scotland.

**DOI:** 10.1038/bjc.1994.424

**Published:** 1994-11

**Authors:** J. N. Newton, A. J. Swerdlow, I. M. dos Santos Silva, M. P. Vessey, D. G. Grahame-Smith, P. Primatesta, D. J. Reynolds

**Affiliations:** Department of Public Health and Primary Care, University of Oxford, Anglia and Oxford Regional Health Authority, UK.

## Abstract

Relatively little is known about the epidemiology of carcinoid tumours in contrast to the extensive information available on their biochemical effects and natural history. Accordingly, we have used cancer registrations in England from 1979 to 1987, and in Scotland from 1980 to 1989, to estimate the incidence of carcinoid tumours in Britain. Age-standardised incidence rates for England, based on 3,382 registrations, were 0.71 (0.68-0.75) for men and 0.87 (0.83-0.91) for women, per 100,000 per year. The equivalent rates for Scotland, based on 639 registrations, were 1.17 (0.91-1.44) for men and 1.36 (1.09-1.63) for women. There was a consistent female excess of carcinoid tumours in the reproductive years, which was reversed after the age of 50. The female excess was most striking for gastrointestinal carcinoid tumours in women aged 15-19 years (F:M ratio = 2.14). The sex differences are probably due in part to incidental diagnosis of carcinoid tumours during abdominal procedures, which are more common in women than men at ages 15-49 years. However, there is some evidence to suggest a true sex difference in incidence, particularly the fact that the sex ratio for thoracic tumours varies with age in a similar way to that for gastrointestinal tumours. Hormonal factors may, therefore, be important in the aetiology of carcinoid tumours.


Br. J. Cancer (1994), 70, 939-942 © Macmillan Press Ltd., 1994

### The epidemiology of carcinoid tumours in England and Scotland

J.N. Newton[1], A.J. Swerdlow[2], I.M. dos Santos Silva[2], M.P. Vessey[3], D.G. Grahame-Smith[4], P. Primatesta[1] & D.J.M. Reynolds[4]

[1]*Unit of Health-Care Epidemiology, Department of Public Health and Primary Care, University of Oxford, Anglia and Oxford Regional Health Authority, Old Road, Headington, Oxford OX3 7LF, UK;* [2]*Epidemiological Monitoring Unit, Department of Epidemiology and Population Sciences, London School of Hygiene and Tropical Medicine, Keppel Street, London WC1 7HT, UK;* [3]*Department of Public Health and Primary Care, University of Oxford, Radcliffe Infirmary, Oxford OX2 6HE, UK;* [4]*MRC Clinical Pharmacology Unit, University Department of Clinical Pharmacology, Radcliffe Infirmary, Oxford OX2 6HE, UK.*

**Summary** Relatively little is known about the epidemiology of carcinoid tumours in contrast to the extensive information available on their biochemical effects and natural history. Accordingly, we have used cancer registrations in England from 1979 to 1987, and in Scotland from 1980 to 1989, to estimate the incidence of carcinoid tumours in Britain. Age-standardised incidence rates for England, based on 3,382 registrations, were 0.71 (0.68-0.75) for men and 0.87 (0.83-0.91) for women, per 100,000 per year. The equivalent rates for Scotland, based on 639 registrations, were 1.17 (0.91-1.44) for men and 1.36 (1.09-1.63) for women. There was a consistent female excess of carcinoid tumours in the reproductive years, which was reversed after the age of 50. The female excess was most striking for gastrointestinal carcinoid tumours in women aged 15-19 years (F:M ratio = 2.14). The sex differences are probably due in part to incidental diagnosis of carcinoid tumours during abdominal procedures, which are more common in women than men at ages 15-49 years. However, there is some evidence to suggest a true sex difference in incidence, particularly the fact that the sex ratio for thoracic tumours varies with age in a similar way to that for gastrointestinal tumours. Hormonal factors may, therefore, be important in the aetiology of carcinoid tumours.

Carcinoid tumours were so named by Oberndorfer in 1907 because they resembled carcinomas but were thought to be of a more benign nature (Grahame-Smith, 1972). Since then the malignant potential of these tumours has been recognised. MacDonald (1956) has suggested that all extra-appendiceal carcinoids should be considered potentially malignant. Carcinoid tumours are the most common tumour of the appendix, the most common gastrointestinal neuroendocrine tumour and the most common form of bronchial adenoma. They may present with non-specific abdominal symptoms, with local symptoms such as haemorrhage or obstruction of the bowel or a bronchus, with evidence of metastasis or with the malignant carcinoid syndrome. This syndrome, first described in 1934, is characterised by facial flushing, bronchoconstriction, episodic diarrhoea and right-sided valvular heart disease (Cassidy, 1934; Grahame-Smith, 1972). It is found in less than 10% of patients with carcinoid tumours in most case series, and generally only after the tumour has metastasised. Many carcinoid tumours are asymptomatic and are found incidentally, for example at appendicectomy or at post-mortem (Berge & Linell, 1976).

Although a number of aspects of carcinoid tumours have been studied in detail, relatively little is known about their epidemiology and no aetiological factors have been established (Basser & Green, 1991). It is difficult to derive incidence rates from the many published case series because of uncertain denominators and potential referral biases. The few population-based studies from the UK (Watson *et al.*, 1989; Woods *et al.*, 1990) have not been large enough to analyse risks by age and sex, and there are few population-based studies from elsewhere (Godwin, 1975; Weiss & Yang, 1987).

### Methods

In both England and Scotland, mortality statistics for carcinoid tumours are not available from routine sources. Cancer deaths in Britain are coded according to the site of the primary tumour not its histological characteristics. Cancer registrations, on the other hand, include information on the site of the primary tumour (if known) and its morphology. Cancer registration in Britain began in 1929 (Stiller, 1993). Coverage has been national in England since 1962 and in Scotland since 1959. Regional registries send data to the national cancer registries at the Office of Population Censuses and Surveys (OPCS) in England and the Information and Statistics Division of the Common Services Agency in Scotland.

### *England*

Cancer registration data were obtained from the OPCS on all carcinoid tumours first registered in 1979-87 in English residents. Although the OPCS also holds Welsh data, these could not be used in this study because they rarely include tumour morphology codes. Registrations of carcinoid tumours were identified using morphology codes 8240-8244 from the International Classification of Diseases for Oncology (ICD-O) (WHO, 1976). The tumours had been given either malignant site codes (ICD-9 140-208) (WHO, 1977) or site codes which indicated that their future behaviour was uncertain (ICD-9 235-238). Before 1979, when ICD-8 (WHO, 1969) was used, tumours of uncertain behaviour were not coded separately from benign tumours and were not recorded by cancer registries. The majority of carcinoid tumours are of uncertain malignancy. This study, therefore, was restricted to the period after 1979 when only tumours specifically reported as benign by the pathologist would not have been registered.

Registrations were subdivided according to tumour site using the ICD-9 classification. Where necessary individual site codes were aggregated as follows: 'gastrointestinal tumours' ICD-9 150.0-154.8 and 235.2; 'thoracic tumours' ICD-9 162.0-165.9, 235.7 and 235.8. The uncertain codes are less specific with respect to site than the malignant codes (see Table I). The natural history of carcinoid tumours is such that the distinction between a malignant tumour and one of potential malignancy is not easily made. On examination of the data, there seemed to be inconsistency among registries in the proportion of cases assigned a malignant, as opposed to an uncertain, site code. Because of this evidence of crossover between the categories we did not analyse malignant tumours separately from those of uncertain behaviour.

As the coding of carcinoid tumours is relatively complex, we conducted a simple telephone survey of registry coding

Correspondence: J.N. Newton
Received 22 February 1994; and in revised form 2 June 1994.

practice for these tumours in England. This showed that similar coding rules existed in all registries. Only two registries did not routinely receive pathology reports on all cancers as well as information from death certificates and other sources. The Thames Registry receives information in electronic format from staff working in the field. In Trent, registrations are generated by hospital records staff from information held on hospital information systems and the registry then obtains pathological information from the clinical notes or from the clinician directly. For the surveillance of carcinoid tumours hospital activity information systems are likely to be less sensitive than a system which includes direct reporting by pathology laboratories.

In order to estimate the completeness of registration of carcinoid tumours using information already to hand, English registries were searched for records of 17 patients known by one of us (D.G.S.) to have had a carcinoid tumour diagnosed histologically. A more formal test of completeness was beyond the scope of this study.

*Scotland*

Data were obtained from the Scottish Common Services Agency on all carcinoid tumours first registered in the years 1980-89 in residents of Scotland. Tumours with malignant or uncertain site codes, ICD-9 140-208 and 235-238, were included. Because the number of tumours was smaller than for England, they were not analysed by site.

*Statistical methods*

Overall rates were directly age standardised using the population of England and Wales in 1981 as a standard for both English and Scottish data. Confidence intervals were calculated using the Poisson approximation to the normal distribution.

### Results

*England*

In the period 1979-87, there were 3,382 carcinoid tumours registered in England. Age-standardised registration rates per 100,000 per year (95% CI) were 0.71 (0.68-0.75) in men and 0.87 (0.83-0.91) in women. The distribution of these tumours by site and behaviour, which was similar in males and females, is given in Table I. Figure 1 shows age- and sex-specific mean annual registration rates for all sites. The incidence rates increased with age in each sex, but declined sharply after 80 years. Incidence rates were consistently greater for females than males throughout the reproductive years. There was a sharp peak in women aged 15-19 years which was not seen in males. After the age of 50 years the sex difference was reversed. Analysis of information from each registry separately (data not reported) showed that these sex differences were not due to anomalous results from any one registry. Figures 2 and 3 show similar data separately for gastrointestinal and thoracic tumours respectively. Gastrointestinal tumours were relatively more common in younger age groups compared with thoracic tumours. The peak of incidence in women aged 15-19 years was only seen for gastrointestinal tumours. The female excess in the reproductive years was apparent in both gastrointestinal and thoracic tumours.

**Figure 1** Age- and sex-specific incidence rates of carcinoid tumours in England, 1979-87, all sites. ■, Male; □, female.

**Figure 2** Age- and sex-specific incidence rates of carcinoid tumours in England, 1979-87, gastrointestinal sites only. ■, Male; □, female.

**Table I** Registered carcinoid tumours in England (1979-87) by site and behaviour

| *ICD-9 code* | *Site* | n | % |
|---|---|---|---|
| *Malignant tumours* | | | |
| 152 | Small intestine | 378 | 11.2 |
| 153 | Colon (including appendix) | 343 | 10.1 |
| 162 | Trachea, bronchus, lung | 203 | 6.0 |
| 197, 198, 199 | Disseminated tumour | 129 | 3.8 |
| 154 | Rectum | 44 | 1.3 |
| 151 | Stomach | 39 | 1.2 |
| | Other sites | 121 | 3.6 |
| *Tumours of uncertain behaviour* | | | |
| 2352 | Stomach, intestines, rectum | 1,544 | 45.7 |
| 2357, 2358 | Thorax | 410 | 12.1 |
| | Other sites | 171 | 5.0 |
| Total | | 3,382 | 100 |

**Figure 3** Age- and sex-specific incidence rates of carcinoid tumours in England, 1979-87, thoracic sites only. ■, Male; □, female.

The test of completeness showed that 15 out of 17 cases independently known to us (see Methods) had been correctly registered in four different registries. One of the remaining two cases was incorrectly registered as a prostatic cancer and the other was not registered.

*Scotland*

In Scotland, 639 carcinoid tumours were registered in the period 1980–89. Age-standardised registration rates per 100,000 per year were 1.17 (0.91–1.44) in men and 1.36 (1.09–1.63) in women. Figure 4 shows age- and sex-specific registration rates for Scotland. Registered incidence rates were generally higher in Scotland than in England, particularly in older age groups. There was no clear peak in women aged 15–19 years, but there was a female excess in the reproductive period.

### Discussion

This investigation is one of a small number of population-based studies of carcinoid tumours. Based on four thousand registrations, it is much larger than previous studies from the UK, none of which have presented data on the age and sex distribution of the tumours. The validity of our results, however, depends on the completeness and accuracy of cancer registrations in England and Scotland. Information on completeness of cancer registration is available from a small number of specific studies (OPCS, 1993), which have shown it to be about 90% in England for certain tumours (Swerdlow *et al.*, 1993). Completeness may vary between tumours, but the histological diagnosis of carcinoid tumour is relatively clear-cut, and likely, therefore, to be reliably reported by pathology laboratories. We have also obtained some direct evidence that registration of carcinoid tumours has taken place with a reasonably high level of completeness and accuracy in parts of England. Our test of completeness was limited to a small number of patients and one geographical area (mainly the Oxford region) but was nonetheless reassuring.

The incidence of carcinoid tumours will have been underestimated if an appreciable number of tumours were not registered because they were reported as benign. American pathologists are said to report less than 2% of all carcinoid tumours explicitly as benign tumours (Godwin, 1975). The clinical impression in the UK is that 'benign' carcinoid tumours are rare and limited to those of the appendix. Although the effect of excluding benign tumours is probably small, the registration of carcinoid tumours would be improved if, in future, all such tumours were registered.

Our estimates of the incidence of carcinoid tumours in England and Scotland can be compared with those for other population-based studies in the US and the UK. The largest

**Figure 4** Age- and sex-specific incidence rates of carcinoid tumours in Scotland, 1980–89, all sites. ■, Male; □, female.

study reported to date used data from cancer registries covering approximately 10% of the population of the US (Godwin, 1975). In that study of 970 cases, age-standardised incidence rates of carcinoid tumour per 100,000 per year were 1.3 in males and 1.6 in females. Thus, Godwin found, as we did, an excess of tumours in women, particularly tumours of the appendix (F:M ratio 3.3:1), which he attributed to a higher rate of laparotomy and incidental diagnosis in women. He also found a female excess for bronchial carcinoids (F:M ratio 1.25:1) as at younger ages in our study. Godwin did not report rates or sex ratios by age group but showed that the average age of patients with appendiceal tumours (36 years) was less than for other sites (50 years for bronchial tumours, 63 years for tumours of the small intestine), which is also consistent with our results. Another American study used cancer registry data but only looked at cancers of the small intestine (Weiss & Yang, 1987). The unadjusted all-ages incidence rate of these tumours was 0.29 per 100,000 per year ($n = 542$). Incidence rates rose sharply after the age of 30 years.

We are aware of only two population-based studies of carcinoid tumours from the UK. In Northern Ireland, from 1970 to 1985, 318 gastrointestinal carcinoid tumours were identified (Watson *et al.*, 1989), giving an incidence rate of 1.3 per 100,000 per year. From 1979 to 1986, 70 cases were recorded by Trent Cancer Registry (Woods *et al.*, 1990). The incidence rate was 0.7 per 100,000 per year.

Our results show a lower incidence rate for carcinoid tumours in England than in Scotland. The rate for England is also lower than Godwin's estimate of incidence in the US. There was, however, some inconsistency in overall registration rates between English regional registries, which suggests that incomplete registration by some registries (as, for example, was the case in Wales) may partially explain the relatively low rate in England.

It has been suggested by Godwin and others that the female excess of abdominal carcinoids, which has also been found in a number of case series (Thompson *et al.*, 1985), is largely explained by diagnostic bias owing to a higher laparotomy rate in women. In support of this it is known, from a study in the Oxford region, that incidental appendicectomy (as opposed to appendicectomy for possible appendicitis) is approximately five times as common in women as in men throughout the reproductive age range (Primatesta & Goldacre, 1994). Admissions for gall stones are also much more common in women than men, the F:M ratio in the age range 15–39 years was 5.7:1, again in the Oxford region (M. Goldacre, personal communication). Furthermore, carcinoids could be discovered during gynaecological procedures including abdominal ultrasound examination. The general female excess of carcinoid tumours in the reproductive age range could, therefore, be due to diagnosis at laparotomy performed for other reasons.

On the other hand, there are reasons to question this assumption. The F:M ratio of appendicectomy rates (for any indication) was only 1.43:1 at 15–19 years (Primatesta & Goldacre, 1994), which was rather less than the F:M ratio of incidence of abdominal carcinoids at the same age in England, which was 2.14. A recently published case series reported that 22 out of 41 appendiceal carcinoid tumours presented with acute abdominal symptoms suggestive of appendicitis (Roggo *et al.*, 1993) and, of the 41 tumours, 33 (80%) were in women. As less than half of the tumours were found incidentally it seems unlikely that the female excess was entirely explained by diagnostic bias. The most convincing evidence is that in our study and elsewhere bronchial carcinoids are also more common in women than men under 50 years. An age-dependent diagnostic bias in favour of females for bronchial tumours seems unlikely. Thus the data suggest that the female excess at reproductive ages, and perhaps also the peak ratio at 15–19, reflects a true sex difference in incidence in addition to a diagnostic bias in favour of females.

Several tumours arising in sites which are not sex specific show marked age-specific sex differences in incidence, e.g.

cancers of the breast, thyroid and descending colon (dos Santos Silva & Swerdlow, 1993), and seem likely to be aetiologically influenced by sex hormones. We have shown that a sex differential is also present for carcinoid tumours, suggesting that endogenous hormones, particularly around the time of puberty, might be important in their aetiology. Oestrogen receptor protein has been identified in carcinoid tumours (Keshgegian & Wheeler, 1980). This finding led to trials of anti-oestrogen therapy in the carcinoid syndrome. There was some early success (Myers *et al.*, 1982), although a later study of 16 patients with metastatic carcinoid tumours showed no benefit from treatment with tamoxifen (Moertel *et al.*, 1984). The epidemiological information presented in this paper suggests that the role of sex hormones as factors in the growth and development of carcinoid tumours should be investigated further.

We thank the Office of Population Censuses and Surveys for supplying, and Andrew Reid for extracting, data on registrations in England, Linda Sharp and Calum Muir of the Scottish Health Service Common Services Agency for providing data and advice on cancer registrations in Scotland, and the staff of the Oxford Cancer Intelligence Unit for performing the telephone survey of registration practice in England. Examination of the English data was funded by the Cancer Research Campaign. The Epidemiological Monitoring Unit is funded by the Medical Research Council. The Unit of Health-Care Epidemiology is funded by the Department of Health and the Anglia and Oxford Regional Health Authority.

### References

BASSER, R.L. & GREEN, M.D. (1991). Recent advances in carcinoids and gastrointestinal neuroendocrine tumours. *Curr. Opin. Oncol.*, **3**(1), 109–120.

BERGE, T. & LINELL, F. (1976). Carcinoid tumours: frequency in a defined population during a 12-year period. *Acta Pathol. Microbiol. Scand.*, **84**, 322–330.

CASSIDY, M. (1934). Abdominal carcinomatosis associated with vasomotor disturbances. *Proc. R. Soc. Med.*, **27**, 220–221.

GODWIN, J.D. (1975). Carcinoid tumors: an analysis of 2837 cases. *Cancer*, **36**, 560–569.

GRAHAME-SMITH, D.G. (1972). *The Carcinoid Syndrome*. Heinemann: London.

KESHGEGIAN, A.A. & WHEELER, J.E. (1980). Estrogen receptor protein in malignant carcinoid tumor. *Cancer*, **45**, 293–296.

MACDONALD, R.A. (1956). A study of 356 carcinoids of the gastrointestinal tract. *Am. J. Med.*, **21**, 867.

MOERTEL, C.G., ENGSTROM, P.F. & SCHUTT, A.J. (1984). Tamoxifen therapy for metastatic carcinoid tumour: a negative study. *Ann. Intern. Med.*, **100**, 531–532.

MYERS, C.F., ERSHLER, W.B., TANNENBAUM, M.A. & BARTH, R. (1982). Tamoxifen and carcinoid tumor. *Ann. Intern. Med.*, **96**, 383.

OFFICE OF POPULATION CENSUSES AND SURVEYS (1993). *Cancer Statistics: Registrations*. HMSO: London.

PRIMATESTA, P. & GOLDACRE, M.J. (1994). Appendicectomy for acute appendicitis and for other conditions: an epidemiological study. *Int. J. Epidemiol.*, **23**, 155–160.

ROGGO, A., WOOD, W.C. & OTTINGER, L.W. (1993). Carcinoid tumours of the appendix. *Ann. Surg.*, **217**(4), 385–390.

DOS SANTOS SILVA, I. & SWERDLOW, A.J. (1993). Sex differences in the risks of hormone-dependent cancers. *Am. J. Epidemiol.*, **138**, 10–28.

STILLER, C.A. (1993). Cancer registration: its uses in research, and confidentiality in the EC. *J. Epidemiol. Commun. Hlth*, **47**(5), 342–344.

SWERDLOW, A.J., DOUGLAS, A.J., VAUGHAN HUDSON, G. & VAUGHAN HUDSON, B. (1993). Completeness of cancer registration in England and Wales: an assessment based on 2,145 patients with Hodgkin's disease independently registered by the British National Lymphoma Investigation. *Br. J. Cancer*, **67**, 326–329.

THOMPSON, G.B., VAN HEERDEN, J.A., MARTIN, J.K., SCHUTT, A.J., ILSTRUP, D.M. & CARNEY, J.A. (1985). Carcinoid tumours of the gastrointestinal tract: presentation management and prognosis. *Surgery*, **98**, 1054–1062.

WATSON, R.G.P., JOHNSTON, C.F., O'HARE, M.M.T., ANDERSON, J.R., WILSON, B.G., COLLINS, J.S.A., SLOAN, J.M. & BUCHANAN, K.D. (1989). The frequency of gastrointestinal endocrine tumours in a well-defined population – Northern Ireland 1970–1985. *Q.J. Med.*, **72**(267), 647–657.

WEISS, N.S. & YANG, C.-P. (1987). Incidence of histologic types of cancer of the small intestine. *J. Natl Cancer Inst.*, **78**, 653–656.

WOODS, H.F., BAX, N.D. & AINSWORTH, I. (1990). Abdominal carcinoid tumours in Sheffield. *Digestion*, **45** (Suppl.1), 17–22.

WORLD HEALTH ORGANIZATION (1969). *International Classification of Diseases*. Eighth revision. WHO: Geneva.

WORLD HEALTH ORGANIZATION (1976) ICD-0. *International Classification of Disease in Oncology*. WHO: Geneva.

WORLD HEALTH ORGNAIZATION (1977). *International Classification of Diseases*, Ninth revision. WHO: Geneva.